# Obesity Paradox in Caucasian Seniors: Results of the PolSenior Study

**DOI:** 10.1007/s12603-019-1257-z

**Published:** 2019-09-30

**Authors:** Monika Puzianowska-Kuznicka, A. Kuryłowicz, D. Walkiewicz, J. Borkowska, M. Owczarz, M. Olszanecka-Glinianowicz, K. Wieczorowska-Tobis, A. Skalska, A. Szybalska, M. Mossakowska

**Affiliations:** 1 Department of Human Epigenetics, Mossakowski Medical Research Centre, PAS, Pawinskiego 5, 02-106 Warsaw, Poland; 2 Department of Geriatrics and Gerontology, Centre of Postgraduate Medical Education, Warsaw, Poland; 3 Department of Pathophysiology, Faculty of Medicine, Medical University of Silesia, Katowice, Poland; 4 Department of Palliative Medicine, Poznan University of Medical Sciences, Poznan, Poland; 5 Department of Internal Medicine and Geriatrics, Jagiellonian University Medical College, Cracow, Poland; 6 PolSenior Programme, International Institute of Molecular and Cell Biology, Warsaw, Poland

**Keywords:** Body mass index (BMI), waist circumference (WC), arm circumference (AC), morbidity, mortality

## Abstract

**Objectives:**

To investigate the influence of overweight and obesity on general performance and mortality in seniors.

**Design:**

Cross-sectional multidisciplinary study on ageing of the Polish population.

**Setting:**

Community-dwelling individuals aged 65 years or older, selected using three-stage stratified, proportional draw.

**Participants:**

4944 Polish Caucasian seniors, aged 65 years or older recruited between October 2007 and October 2010.

**Measurements:**

All study subjects underwent measurement of body mass index (BMI), waist circumference (WC), and arm circumference (AC). The physical and cognitive performance was evaluated using the Katz Activities of Daily Living (ADL) score and Mini-Mental State Examination (MMSE), respectively. Morbidity data were obtained from a medical questionnaire. Mortality data were obtained from the Population Register of Poland between October 2015 and October 2018.

**Results:**

Increasing age was associated with a decreased prevalence of obesity (all p<0.001). Higher BMI, WC and AC values were associated with higher ADL and MMSE scores (all p<0.001). On multivariate analysis, all three body measurements in women remained independent predictors of the ADL score (BMI p=0.002, WC p=0.005, AC p<0.001) and MMSE score (p<0.001, p=0.003, p<0.001). In men, physical functioning was associated with AC (p=0.003), and cognitive status was associated with AC (p<0.001) and BMI (p=0.013). There was no association between general obesity, abdominal obesity, or AC with several aging-related adverse conditions. Kaplan-Meier survival curves showed that overweight and obesity were associated with the lowest mortality. On multivariate analysis, BMI and AC values remained independent predictors of mortality. In successfully aging individuals, neither BMI, WC, nor AC remained such predictors.

**Conclusions:**

Overweight and obesity in Caucasian seniors are not associated with deterioration of physical and cognitive function or with increased mortality.

**Electronic Supplementary Material:**

Supplementary material is available for this article at 10.1007/s12603-019-1257-z and is accessible for authorized users.

## Introduction

Obesity is a major public health problem. Over the last four decades, the global prevalence has increased from 3.2% to 10.8% in men and from 6.4% to 14.9% in women, with the upward trend persisting (1). Significant differences in the incidence of obesity between populations result from genetic disparities, dietary and physical activity patterns, and differences in socioeconomic status (2–4). Epidemiological studies suggest that, even though the prevalence of obesity increases with age, this trend stops or even reverses in the age group older than 60 to 70 years (5, 6). This view, however, is not held by all researchers (7).

Obesity, through its metabolic and endocrine effects, significantly increases the incidence of many conditions, including type 2 diabetes, cardiovascular disease, and cancer (8). It also increases the risk of physical disability with a consequent reduction in quality of life (9), and its associated complications increase mortality (10). In older age groups, however, excess weight seems to have no negative effect on mortality (11, 12). Moreover, it is suggested that in long-lived humans, overweight and obesity might have a protective effect, an observation described as the “obesity paradox” (13–16).

Another public health problem that must be urgently addressed is the aging of the population. According to the estimation of the World Health Organization, in the middle of the twenty-first century, almost all European and North American countries will have more than 30% of citizens 60 years of age or older (17). The phenotype of aging varies greatly between individuals. Such differences are due in part to genetic background (18, 19), but most reflect the long-term influences of environment and lifestyle on gene activity and cellular metabolism (20).

To reduce the burden imposed by aging and aging-related disease on health care systems and to improve quality of seniors’ life, it is necessary to undertake actions ensuring not only longer, but also healthier lives. One of the first steps in this undertaking is an appreciation of how basic measurements of body mass and composition are associated with physical and cognitive performance, morbidity, and mortality in seniors and oldest-old individuals.

## Materials and Methods

### Study design

The study was conducted as a part of the PolSenior project — a multicenter study focused on elderly subjects, implemented between October 2007 and October 2010. The project was coordinated by the International Institute of Molecular and Cell Biology (IIMCB) in Warsaw, Poland. The Study Scientific Board formulated the final study protocol, elaborated survey questions, selected laboratory parameters for analysis, prepared instructions for nurse trainers, monitored study flow, controlled the work of the Central Laboratory and regulated of access to the Central Database. Independently, an International Scientific Committee composed of experts in the field of geriatrics, gerontology and epidemiology was created to oversee the design and conduct of the study.

The study was composed of four parts performed by nurses: (i) questionnaire survey; (ii) comprehensive geriatric assessment, including selected scales and tests; (iii) blood pressure and anthropometric measurements; (iv) blood and urine sample collection. Additionally, a detailed medical examination by geriatricians was performed in those participants who have accepted the proposal of further geriatric assessment.

The 466 interviewers were recruited by the PBS DGA company, an agency with expertise in research projects for private and public sectors. They were active nurses who worked within local communities and were trained for the purpose of the PolSenior project. The interviewer visited each study participant three times in order to complete the medical and socioeconomic questionnaire and to collect blood and urine samples (21, 22).

The detailed information about the PolSenior project is available online at http://polsenior.iimcb.gov.pl/en.

### Sampling method

Initially, the PolSenior program was planned to include 5,950 participants divided into six age groups of equal size and gender distribution (65–69, 70–74, 75–79, 80–84, 85–89 and ≥90 years), and a reference group consisting of subjects who were about to enter old age (55–59 years old). The responders were randomly recruited in bundles in a stratified, proportional three-stages draw. In the first stage local administrative units, including urban, rural, and urban-rural municipalities were identified. Towns and cities were divided into five groups, depending on size (≤ 20,000, > 20,000–50,000, > 50,000–200,000, 200,000–500,000, > 500,000 residents). In the second stage, streets in urban municipalities, and villages in rural municipalities were drawn. In the third stage of the draw, using the national database run by the Ministry of Internal Affairs and Administration, respondents were selected based on the Universal Electronic System for Registration of the Population (PESEL) (21). Out of 15,574 randomly selected addresses, 13,376 residents were eligible for the study and 5,695 respondents took part in the project. In this group, 4,979 individuals were ≥65 years old (Supplementary Figure 1). Details regarding study design and population were previously published (21).

**Figure 1:**
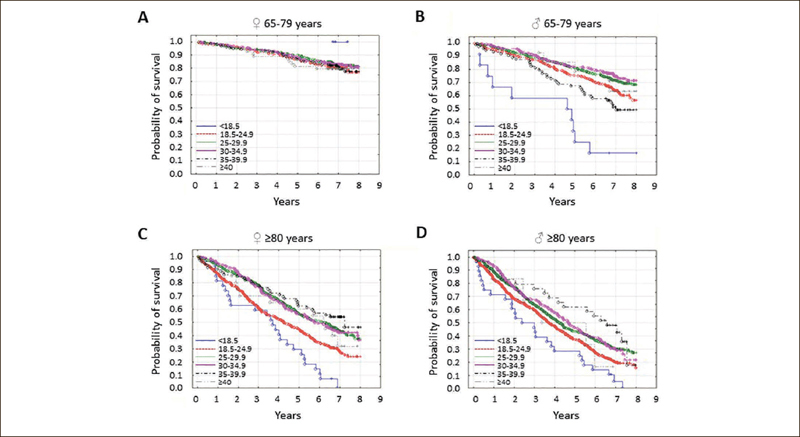
The probability of survival in seniors by body mass index [BMI] category

Study participants were stratified according to age and sex. In younger seniors [65–79 years], survival in women [A] was independent of BMI [p=0.82]. In younger senior men [B], older senior women [C], and senior men [D,] survival was associated with BMI [all p<0.001]. The BMI categories were: <18.5 kg/m2 [malnutrition], 18.5 to 24.9 kg/m2 [normal body weight], 25.0 to 29.9 kg/m2 [overweight], 30 to 34.9 kg/m2 [moderate obesity], 35.0 to 39.9 kg/m2 [severe obesity], and ≥40 kg/m2 [morbid obesity].

### Study participants, anthropometric measurements and medical questionnaires

4,944 Caucasian seniors (2390 women, 2554 men) out of 4,979 participants of the PolSenior program aged ≥65 years were included in the present study. The remaining 35 individuals were not included due to the lack of body measurements. All anthropometric measurements and questionnaire data were obtained between October 2007 and October 2010. The physical examination included calculation of body mass index (BMI, 4624 individuals) and measurement of waist circumference (WC, 4724 individuals) and arm circumference (AC, 4904 individuals).

For the purposes of the current study, aging-associated adverse conditions included cardiovascular disease (e.g., heart failure, myocardial infarction, stroke), type 2 diabetes mellitus, chronic kidney disease (estimated glomerular filtration rate <60 mL/min), chronic obstructive pulmonary disease, cancer (skin cancers other than melanoma were not accounted for), a Mini-Mental State Examination (MMSE) score less than 24, and a Katz Activities of Daily Living (ADL) score less than 5. Survival data were obtained from the Population Register of Poland 8 years after recruitment into the study (from October 2015 and October 2018). At the moment of access to the register, 2392 individuals had died. The total number of person-years of observation was 261 594.

### Mini-Mental State Examination and Activities of Daily Living scores

Cognitive function was assessed using the MMSE preceded by a rough assessment of the participant’s hearing and vision (23). The study participants were divided into the following groups: normal cognition, minimal cognitive impairment, mild cognitive impairment, moderate cognitive impairment, and severe cognitive impairment (MMSE scores 28–30, 24–27, 20–23, 10–19, and <10 points, respectively). Physical performance was assessed using the ADL scale (24). The study participants were divided into independent, partially dependent, and totally dependent groups (ADL scores 5–6, 3–4, and 0–2 points, respectively).

### Statistical analysis

Statistical analyses were performed using Statistica software, version 10 (Statsoft Inc., Tulsa, OK, USA) and the R program (R Foundation for Statistical Computing, Vienna, Austria). Due to the skewed distribution of values for BMI, WC, and AC, variables were calculated as the median (first quartile, third quartile). The significance of the relation between analyzed factors was tested using nonparametric Kruskal-Wallis one-way analysis of variance (ANOVA) and rank-based nonparametric analysis of covariance (ANCOVA) for multifactorial analyses. We used Spearman’s rank correlation coefficient (rho) as a measure of correlation between parameters. A Kaplan-Meier plot was used to present survival curves, which were compared using the log-rank test. The Cox proportional hazards model was used for univariate and multivariate survival analyses, with age as one of the covariates. Proportional-hazards assumption was tested using Schoenfeld residuals. For all statistical analyses, the level of significance was set at 0.05.

### Ethical and legal issues

The study was approved by the Bioethics Commission of the Medical University of Silesia in Katowice. Each respondent or his/her legal guardian signed informed consent and was assigned a unique identification number to ensure confidentiality of personal data. The data were anonymously stored in a Central Database.

## Results

### Body measurements

Using BMI 30 kg/m^2^ or greater as the definition of obesity, we found that 31.66% of study participants were obese and that there were more obese women than men (38.4% vs 25.5%, p<0.001). In addition, using a WC measurement of 80 cm or greater in women and 94 cm or greater in men as the cutoff for defining abdominal obesity, we found that 80.6% of all study participants had a WC measurement above the cutoff. Women were more commonly affected than men (89.2% vs 72.6%, p<0.001). We found that the percentage of obese individuals decreased with age in the entire cohort (Table 1) and in women and men separately (all p<0.001). Similarly, the percentage of individuals with a high WC decreased with age in the entire cohort (Table 1) and each sex separately (all p<0.001). Notably, the median BMI, WC, and AC measurements systematically decreased with age in the entire cohort (Table 2) and both sexes separately (all p<0.001).

**Table 1:** Obesity in >65 years old seniors divided into 5-year age groups

	**BMI [≥30 kg/m** ^**2**^ **]**		**WC [W: ≥80 cm, M: ≥94 cm]**	
**Age [years]**	**N**	**%**	**N**	**%**
65–69	770	40.8	776	84.8
70–74	900	41.2	906	85.8
75–79	812	33.6	828	81.3
80–84	749	31.8	758	83.8
85–89	773	22.9	802	78.6
≥90	620	14.7	654	66.1

N: number of study participants belonging to a given age group, %: percentage of obese study participants within age group, BMI: body mass index, WC: waist circumference, W: cut-off value for women, M: cut-off value for men.

**Table 2:** Median BMI, waist circumference and arm circumference in ≥65 years old seniors divided into 5-year age groups

**Age [years]**	**BMI [kg/m** ^**2**^ **]** ^*^	**WC [cm]** ^*^	**AC [cm]** ^*^
65–69	29.0 (25.8, 32.5)	100.0 (91.0, 108.0)	30.0 (28.0, 33.0)
70–74	29.0 (25.7, 32.4)	100.0 (92.5, 110.0)	30.0 (28.0, 32.5)
75–79	28.1 (25.3, 31.4)	99.0 (90.8, 107.0)	29.0 (27.0, 31.5)
80–84	27.7 (24.9, 30.8)	99.0 (91.0, 108.0)	28.2 (26.0, 31.0)
85–89	26.6 (23.7, 29.6)	98.0 (89.0, 105.0)	27.5 (25.0, 30.0)
≥90	25.1 (22.2, 28.4)	93.0 (85.0, 102.0)	26.0 (23.5, 28.0)

^*^: median (1st quartile, 3rd quartile), BMI: body mass index, WC: waist circumference, AC: arm circumference.

### Association of body measurements with physical and cognitive function

Evaluation of physical function showed that higher median BMI, WC, and AC values were associated with higher ADL scores (Table 3, all p<0.001). Both AC and WC were predictors of physical functioning in both sexes, while BMI was a predictor only in women. Next, we performed multivariate analysis for age, smoking status, and BMI or WC or AC values. Independent variables were selected based on literature (25–27). We found that all three body measurements remained independent predictors of ADL score in women (p=0.002, p=0.005, p<0.001, respectively). In men, only the AC was an independent predictor of ADL score (p=0.003).

**Table 3:** Obesity and the Activities of Daily Living score

	**BMI [kg/m** ^**2**^ **]**		**WC [cm]**		
**ADL [points]**	**<30**	**≥30**	**W: <80, M: <94**	**W: ≥80, M: ≥94**	
5–6	67.7%	32.3%	18.6%	81.4%	
3–4	74.5%	25.5%	26.3%	73.7%	
0–2	78.7%	21.3%	28.0%	72.0%	
**ADL [points]**	**BMI [kg/m** ^**2**^ **]** ^*^		**WC [cm]** ^*^		**AC [cm]** ^*^
5–6	27.8 (24.8, 31.2)		99 (90, 107)		29.0 (26.5, 31.5)
3–4	26.0 (22.7, 30.2)		97 (89, 107)		26.5 (24.0, 30.0)
0–2	25.4 (21.7, 28.6)		93 (83, 105)		25.0 (23.0, 28.5)

^*^: median (1st quartile, 3rd quartile), ADL: Activities of Daily Living, BMI: body mass index, WC: waist circumference, AC: arm circumference, W: cut-off value for women, M: cut-off value for men; %: percentage of the studied population

Evaluation of cognitive function showed that higher median BMI, WC, and AC values were associated with higher MMSE scores (Table 4, all p<0.001). Both AC and WC were predictors of cognitive performance in women and men, but BMI was only a predictor in women. On multivariate analysis for age, smoking status (27–29), and BMI or WC or AC values, all three body measurements remained significant predictors of cognitive performance in women (p<0.001, p=0.003, p<0.001, respectively). In men, only BMI (p=0.013) and AC (p<0.001) were independent predictors.

**Table 4:** Obesity and the Mini Mental State Evaluation score

	**BMI [kg/m** ^**2**^ **]**		**WC [cm]**		
**MMSE [points]**	**<30**	**≥30**	**W: <80, M: <94**	**W: ≥80, M: ≥94**	
28–30	63.5%	36.5%	16.3%	83.7%	
24–27	68.4%	31.6%	18.2%	81.82%	
20–23	69.2%	30.8%	19.7%	80.3%	
10–19	77.5%	22.5%	29.1%	70.9%	
<10	84.0%	16.0%	28.9%	71.2%	
**MMSE [points]**	**BMI [kg/m** ^**2**^ **]** ^*^		**WC [cm]** ^*^		**AC [cm]** ^*^
28–30	28.4 (25.6, 31.7)		100 (91.0, 108.0)		29.5 (27.0, 32.0)
24–27	27.8 (24.9, 31.2)		99.0 (91.0, 107.5)		29.0 (26.5, 31.5)
20–23	27.3 (24.0, 30.9)		98.0 (89.5, 106.0)		28.0 (25.5, 31.0)
10–19	26.2 (22.8, 29.1)		96.0 (87.0, 104.0)		27.0 (24.0, 29.5)
<10	25.2 (22.5, 27.6)		92.0 (84.0, 102.0)		25.0 (23.0, 28.0)

^*^: median (Q1, Q3), ADL: Activities of Daily Living, BMI: body mass index, WC: waist circumference, AC: arm circumference, W: cut-off value for women, M: cut-off value for men: %: percentage of the studied population

### Association of body measurements with multimorbidity

There was no association between general obesity, abdominal obesity, or AC with several aging-related adverse conditions. However, obesity and abdominal obesity were associated with the incidence of some diseases. In both men and women, a BMI 30 kg/m^2^ or higher was associated with a higher frequency of diabetes (both p<0.001). In men, a BMI 30 kg/m^2^ or higher was associated with a lower incidence of cancer (p=0.03).

Abdominal obesity was associated with a higher frequency of diabetes (p<0.001) in both men and women (both p<0.001) and of cardiovascular disease (p<0.001) in both men and women (p<0.001 and p=0.02, respectively). Abdominal obesity was associated with a lower frequency of cancer in men (p=0.04).

Increasing quartiles of AC were associated with an increasing frequency of cardiovascular disease in women (p=0.006), increasing frequency of dementia and diabetes in both sexes together and separately (all p<0.001), and a decreasing frequency of cancer in men (p=0.006) and kidney insufficiency in women (p=0.002).

### Association of body measurements with mortality

We used Kaplan-Meier curves to verify the effect of body weight on mortality in seniors. We assessed individuals 65 to 79 years and those 80 years or older separately, with additional stratification by sex. In the younger senior men, a BMI of 25.0 to 34.9 kg/m^2^ was associated with the lowest mortality, while underweight (BMI <18.5 kg/m^2^) was associated with the highest mortality (p<0.001). In women, however, there was no such association (Figure 1). In individuals of both sexes aged 80 years or greater, a BMI of 25.0 to 39.9 kg/m^2^ was associated with the lowest mortality, while underweight and normal weight (BMI ≤24.9 kg/m^2^) were associated with the highest mortality (Figure 1, p<0.001 for women and men).

**Figure 2:**
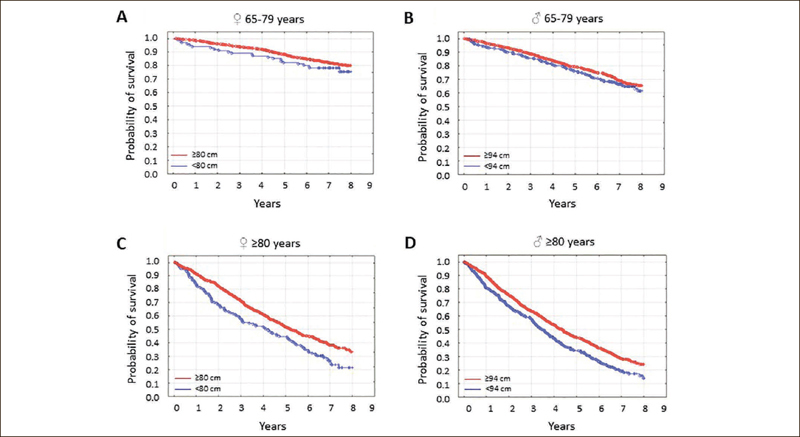
The probability of survival in seniors by waist circumference category

Abdominal obesity was not associated with mortality in younger seniors of either sex, but the absence of abdominal obesity was associated with higher mortality in both women and men aged 80 years or greater (Figure 2, p=0.001 and p<0.001, respectively).

Study participants were stratified according to age and sex. In younger senior [65–79 years] women [A] and men [B], survival was independent of waist circumference [p=0.19, p=0.12]. In older senior women [C] and men [D], it was associated with WC [p=0.001, p<0.001]. The waist circumference categories were: ≤80 or ≥80 cm for women, and <94 or ≥94 cm for men.

**Figure 3:**
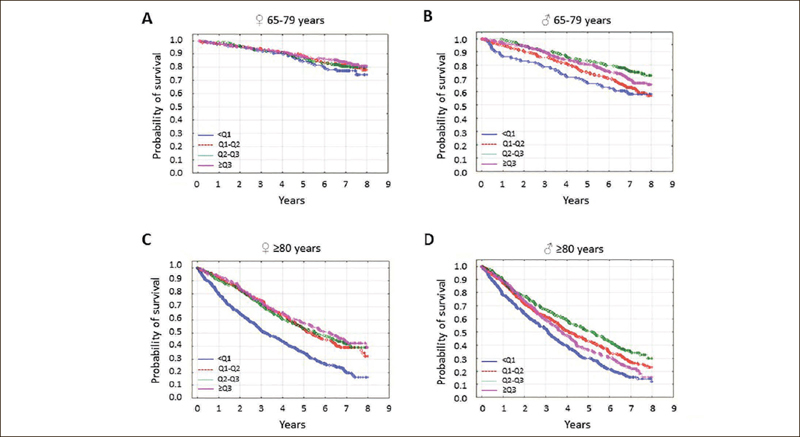
The probability of survival in seniors by arm circumference quartile

In the younger seniors, the lowest AC quartile was associated with the highest mortality in men only; in older study subjects, a low AC was associated with the highest mortality in both sexes (Figure 3; both p<0.001).

Study participants were stratified according to age and sex. In younger senior [65–79 years] women [A], survival was independent of arm circumference quartile [p=0.37]. In younger elderly men [B], older senior women [C], and senior men [D], survival was associated with arm circumference quartile [all p<0.001]. Arm circumference values were divided into quartiles because of the lack of standardized cutoff values.

Multivariate analysis for age, sex, number of aging-associated diseases, smoking status and BMI or WC or AC, we used normal BMI values as a reference. In women, BMI remained an independent predictor of mortality, with the lowest hazard ratio (Hr) seen with a BMI of 35.0 to 39.9 kg/m^2^ (HR 0.6, 95% confidence interval [95%CI], 0.43–0.84, p=0.002). Other independent predictors of mortality in women were age and the number of aging-related diseases. In men, BMI remained an independent predictor of mortality, with the lowest hazard ratio seen with a BMI of 30 to 34.9 kg/m^2^ (HR 0.81, 95%CI, 0.66–0.99, p=0.037). In addition, age, number of aging-related diseases, and smoking status also remained independent predictors of mortality.

When WC values were divided into a reference group without abdominal obesity and a group with abdominal obesity, age and the number of aging-related diseases were independent predictors of mortality in both sexes, and smoking status was an additional independent predictor in men. Abdominal obesity was not an independent predictor of mortality.

The reference value for AC measurement was established as the first quartile. Increasing quartiles were independent predictors of decreasing mortality in both sexes (women: Q1–Q2: HR 0.82 [95%CI 0.67–0.99], p=0.048; Q2–Q3: HR 0.73 [95%CI 0.59–0.90], p=0.004; ≥Q3: HR 0.68 [95%CI 0.54–0.85], p<0.001 and men: Q1–Q2: HR 0.78 [0.66, 0.93], p=0.005; Q2–Q3: HR 0.66 [95%CI 0.54–0.80], p<0.001; ≥Q3: not significant). In addition, age and the number of aging-associated diseases were predictors of mortality in both sexes, while smoking status was an independent predictor only in men.

Finally, we evaluated mortality in successfully aging seniors in relation to BMI, WC, or AC adjusted for age, sex, and smoking status. We found that in women, only age remained an independent predictor of mortality. In men, the independent predictors were age and smoking status.

## Discussion

In young and middle-aged individuals, excess weight is among the major risk factors for the development of cardiometabolic and other diseases and for early death (8, 10). Yet even though body fat increases with age, especially in the abdominal area, excess weight in seniors may not be as damaging as previously thought (13–16). In the present work, we show that a higher BMI, WC, and AC are associated with better physical and cognitive functioning in seniors, even when age, sex, and smoking status are taken into account. Even though both general obesity and abdominal obesity are positively correlated with the incidence of diabetes and cardiovascular disease, they do not correlate with the number of aging-related adverse conditions. Moreover, obese male study subjects have a lower frequency of past and present cancers than nonobese men, even though obesity is reportedly a risk factor for the development of some cancers (30). We acknowledge that the significance of this finding could result from the low number of cancer diagnoses in our study cohort.

In the entire cohort, not preselected according to the health and functional status, being aged 80 years or greater with overweight or obesity is associated with the best survival, even when age, number of aging-related adverse conditions, and smoking status are taken into account. This result suggests that, during aging complicated by such conditions, a higher body mass can exert a protective effect. It remains to be elucidated which of these conditions, if any, are less detrimental in the presence of obesity or whether a protective effect of obesity relies on general effects. Underweight is associated with the worst survival, and this condition can result from the terminal phase of cancer. In our study group, however, a current cancer diagnosis had no significant effect on mortality in underweight individuals compared with the other weight groups combined (data not shown). Thus, it is possible that underweight reflected inadequate nutrition and frailty or the presence of other life-threatening conditions (31, 32).

In successfully aging seniors, we observed no positive or negative association of body measurements with mortality. This suggests that, in such individuals, overweight is not as detrimental as it is in younger humans, but neither it is protective. Some authors claim that the “obesity paradox” might, in fact, be a result of selection bias and methodological errors such as omitting important confounders in the analysis (e.g., smoking status, adipose tissue distribution) (33, 34). In our work, however, study participants were not preselected, and we analyzed not only the BMI, but also the WC, AC, and smoking status. The fact that we saw no negative effect of overweight and obesity on mortality in successfully aging seniors supports the “obesity paradox” hypothesis.

Presumably, the reasons why general and abdominal obesity are not detrimental in seniors, especially in the oldest-old, are numerous. We limit our discussion to the most likely candidates. First, although calorie restriction is the best-known effective method of extending lifespan and healthspan, there is a growing body of evidence indicating that diet composition is at least equally important (35, 36). Therefore, in the elderly, overweight or obesity might be associated with a better supply of vitamins, microelements, plant polyphenols, and other nutrients that ensure the proper functioning of molecular pathways involved in the regulation of the rate and phenotype of aging. In seniors, especially the oldest-old, low body weight is usually not a result of intentional caloric limitation but rather results from malnutrition or severe disease (37). This is a plausible explanation for our observation that the highest mortality occurs in those who are underweight.

Second, not all obesity is associated with adipose tissue dysfunction, in which macrophage infiltration is seen, and excess inflammatory mediators and adipokines are produced. Individuals without this complication are known as “metabolically healthy obese” (38, 39).

Third, in men and postmenopausal women, peripheral synthesis is the main source of estrogen, and adipose tissue is one of the sites of peripheral synthesis (40, 41). The obese, with large deposits of adipose tissue, produce more estrogen than normal-weight and underweight individuals. Consequently, the estrogen produced by adipose tissue located nearby and within various organs may exert protective effects on these organs through the local autocrine and paracrine action (42). A larger amount of estrogen also enters the circulation in these patients and can positively affect distant tissues (41).

Finally, overweight and obese individuals are often taking medicine for cardiometabolic disease: the use of metformin, statins, and acetylsalicylic acid is more common than in their same-age non-overweight or non-obese counterparts. These medications slow the rate of aging and decrease the risk and severity of already existing disease, thus increasing the length of life (43, 44).

Our study has some limitations. The first one regards the representativeness of the studied group. The project planned an equal number of respondents in each age group with equal gender distribution. This assumption allowed for a precise analysis of the examined factors in the oldest groups, which was a priority of the PolSenior study. Therefore, the structure of the studied population did not reflect the demographic structure of the senior population, as the oldest age groups were overrepresented.

Similarly, the inclusion of equal numbers of men and women resulted in overrepresentation of men. Therefore, to achieve the representativeness of the studied group, the weighting adjustment method was applied (45). Another limitation is a low response rate (42.58%) that might have led to some selection bias, however, the response rate was comparable in all age cohorts and for both sexes (21).

Next, other methods of assessing obesity, such as bioelectric impedance or dual-energy X-ray absorptiometry, are much better than BMI, WC, and AC because they allow for an estimation of body composition. However, in everyday practice, using a measuring tape and scale is fast, cost-free, and can be easily repeated during subsequent checkups. Therefore, to allow physicians to estimate body mass quickly, and to compare the results to the data of other reports, BMI and WC are typically used. However, BMI and WC measurements might be incorrect, especially in the oldest-old, due to spinal deformation (46). Another limitation is that we do not know the trajectory of obesity in our study subjects and therefore cannot evaluate how long-term and short-term obesity affect the phenotype of aging.

The strength of this work relies on equally-sized 5-year age cohorts containing a similar number of women and men, allowing for a reliable analysis of the oldest study subjects and accurate comparison with other age groups and with the reports of other authors. We had access to detailed medical histories, current health status reports, and the results of the ADL and MMSE tests performed by trained nurses. We are confident that our analysis of the association of body mass with clinical data generated reliable results.

In conclusion, our data show that overweight and obesity in Caucasian seniors are associated neither with a worsening of physical and cognitive function nor with increased mortality. Our data, and that of other authors suggest that senior individuals might function differently than young and middle-aged individuals. Therefore, consideration should be given to the creation of other body mass-related recommendations for seniors.

## Supplementary Material

Supplementary material, approximately 137 KB.
