# Supplementary figures and images for: Obesity Paradox in Caucasian Seniors: Results of the PolSenior Study
Source: J Nutr Health Aging. 2019 Sep 30;23(9):796–804. doi: 10.1007/s12603-019-1257-z (PMC6800404; doi:10.1007/s12603-019-1257-z)

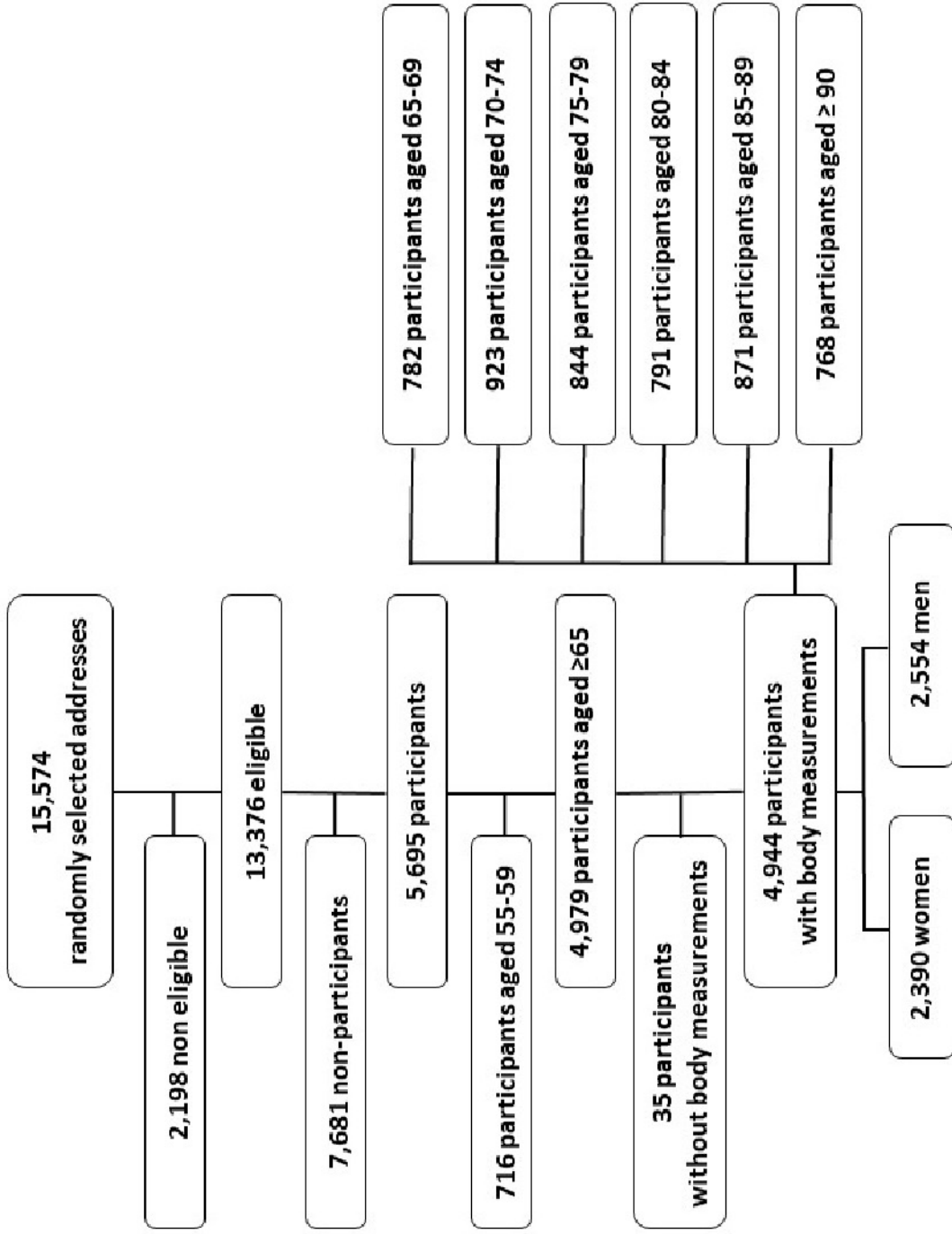

Supplement: Supplementary file 1 — Supplementary material, approximately 137 KB. [file mmc1.pdf]
