# Supplementary material for: Obesity Paradox in Caucasian Seniors: Results of the PolSenior Study
Source: J Nutr Health Aging. 2019 Sep 30;23(9):796–804. doi: 10.1007/s12603-019-1257-z (PMC6800404; doi:10.1007/s12603-019-1257-z)
Supplement: Supplementary file 1 [file 12603_2019_1257_MOESM1_ESM.pdf]

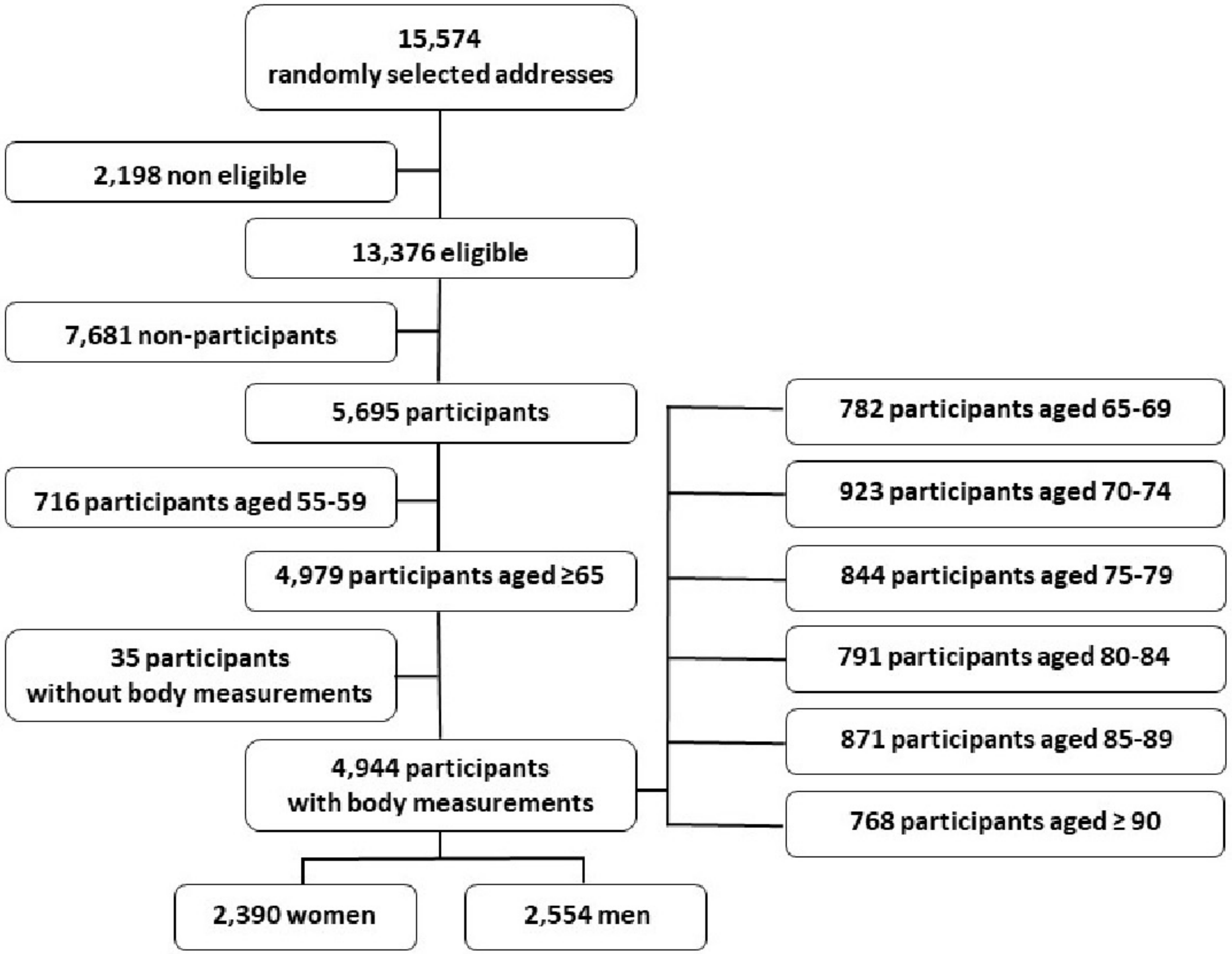
15,574
randomly selected addresses
2,198 non eligible
13,376 eligible
7,681 non-participants
5,695 participants
716 participants aged 55-59
4,979 participants aged ≥65
35 participants
without body measurements
4,944 participants
with body measurements
782 participants aged 65-69
923 participants aged 70-74
844 participants aged 75-79
791 participants aged 80-84
871 participants aged 85-89
768 participants aged ≥ 90
2,390 women
2,554 men
